# The introduction and evaluation of novel decellularized extracellular matrix/gellan gum bioprinting scaffolds for cartilage tissue engineering

**DOI:** 10.1038/s41598-025-11559-w

**Published:** 2025-09-30

**Authors:** Melika Sahranavard, Ali Zamanian, Aliasghar Behnamghader, Mostafa Shahrezaee

**Affiliations:** 1 Biomaterials Research Group, Department of Nanotechnology and Advanced Materials, Materials and Energy Research Center, Karaj, Iran; 2 Trauma Research Institute, Tehran, Iran

**Keywords:** dECM, Gellan gum, 3D Bioprinting, Bioink, Cartilage tissue engineering, Biomedical engineering, Biomaterials

## Abstract

Cartilage demonstrated limited self-regeneration; and there is a need for developing new compounds. Here, gellan gum was selected due to its hydrophilicity, biocompatibility, and native cartilage environmental resemblance, and cartilage decellularized extracellular matrix (dECM) was added (GG/dECMb) to improve the cellular interactions. The decellularization was performed using freeze-thaw cycles and sodium dodecyl sulfate and a hematoxylin-eosin and Bradford assays showed successful decellularization with low extracellular matrix damage. The GG/dECMb compound was formulated, and the gellan gum was considered as the control (GGb). The rheological evaluations demonstrated the shear-thinning and bioprinting capability, while the GG/dECMb had a lower cross-linking degree (5.04 ± 0.79%) in comparison to GGb (6.65 ± 0.48%). Both bioinks were successfully bioprinted. The mechanical test demonstrated the GG/dECMb had a damping feature, which is essential for cartilage regeneration. Furthermore, it has a higher hydrophilic nature (44.27 ± 6.0° contact angle), swelling ratio, and biodegradation ratio in comparison to GGb. The cellular tests confirmed the high capability of GG/dECMb dried scaffolds in cell viability based on the cell viability test (97.41 ± 1.02%) and live/dead assays. The Alcian blue staining proved the glycosaminoglycans deposition and cartilage differentiation of GG/dECMb. Therefore, it seems that GG/dECMb can be effective in cartilage regeneration, although needs further in-vivo studies in the future.

## Introduction

The cartilage extracellular matrix (ECM) primarily consists of glycosaminoglycans (GAGs) and proteoglycans (PGs), which contribute to the tissue’s biomechanical properties by forming a slippery surface that reduces friction and absorbs mechanical shocks, thereby protecting bones from cracking^1^.

Cartilage-related disorders, including osteoarthritis, affect over 90 million individuals annually in the United States, according to epidemiological estimates^2^. Although these defects are most common in the elderly population, cartilage injuries in young individuals can impair their mobility^3^. With the aging of the population all around the world, it is expected that cartilage injuries become more widespread^4^.

Cartilage is a connective tissue with limited self-regeneration capacity due to its avascular nature and scarcity of regeneration cells^5^. Numerous treatment approaches, such as debridement, drilling, arthroscopic surgery, and autologous/xenograft transplantation surgery, have been employed for cartilage regeneration, but these repairing strategies can’t meet the complete cartilage function restoration^6^. In this regard, there is an urgent need to find innovative therapeutic strategies and methods for cartilage regeneration.

Cartilage tissue engineering has emerged as a promising approach and demonstrated a high potential in cartilage defect repair. Scaffolds play a crucial role in cartilage tissue engineering by providing structural support and guiding cell behavior. There are various scaffold fabrication methods and among these techniques, 3D bioprinting has gained a lot of attention due to its advantages in comparison to traditional scaffold fabrication methods, such as high precision, personalization, reprinting capability, and mimicking the anatomical structure of the native tissue^7,8^. This technology provides layer-by-layer deposited patient-specific constructs based on the pre-designed computer-aided model^9,10^.

In addition to printing precision, the choice of bioink materials is crucial for achieving desirable mechanical and biological properties in 3D-bioprinted scaffolds. Gellan gum is a linear, anionic biocompatible polysaccharide that has been proposed for cartilage tissue engineering applications due to its biocompatibility, biodegradability, mechanical properties, and easy gelation. Its gelation occurs through ionotropic mechanisms in the presence of cations such as Ca²⁺, Mg²⁺, Na⁺, and K⁺^11,12^. Compared to other common hydrogels in cartilage tissue engineering, such as Gelatin Methacryloyl, which requires methacryloylization of gelatin and ultraviolet irradiation for crosslinking and may damage cells, gellan gum can easily gels under physiological conditions without the need for irradiation^13^. Additionally, gellan gum also provides an environmental resemblance of native cartilage than alginate, making gellan gum a safer and simpler choice for sensitive biological applications such as cell culture or bioprinting^14^.

Despite the gellan gum promising properties, its lack of cell binding sites can restrict cell adhesion, migration, and proliferation. Therefore, many studies focused on combining gellan gum with other materials with higher cell adhesion properties, such as chitosan^15^ silk fibroin^14^ chondroitin sulfate^16^ and other biocompatible materials.

Here, incorporating decellularized extracellular matrix (dECM) into gellan gum was suggested for improving the bioactivity and cellular interactions. Each tissue has a unique ECM that provides mechanical and chemical cues for the ideal performance of that tissue. dECM is a rich reservoir of natural molecules, including proteins and growth factors, which is achieved by removing the cells from the tissue while preserving the components of ECM^17,18^. Pure dECM shows low bioprintability and cannot provide sufficient mechanical properties for direct use in tissue engineering^19^. It seems the addition of dECM to gellan gum hydrogel could show a synergistic effect and improve the bioprintability of dECM simultaneously enhancing the cellular behavior of gellan gum. To our knowledge, this is the first study to use a combination of urea-extracted dECM from cartilage tissue and gellan gum to formulate an optimized bioink for 3D bioprinting applications in cartilage tissue engineering. This innovative combination aims to overcome the limitations of gellan gum and pure dECM, synergistically improving printability and enhancing cellular response.

Therefore, in this study, cartilage tissue was decellularized, and extracted dECM was prepared after confirmation of the successful decellularization process. Urea extraction is one of the common dECM extraction methods. Urea is a chaotropic agent that disrupts hydrogen bonding^20^ and provides high GAG content dECM for further investigations. Therefore, after dECM urea extraction preparation, a novel and optimized gellan gum/dECM bioink formulation was developed, and utilized in a 3D bioprinting procedure to prepare scaffolds. The 3D bioprinted gellan gum/dECM scaffolds were evaluated for their suitability in cartilage tissue engineering applications.

## Results and discussion

### Hematoxylin and Eosin (H&E) observations

**Fig. 1:**
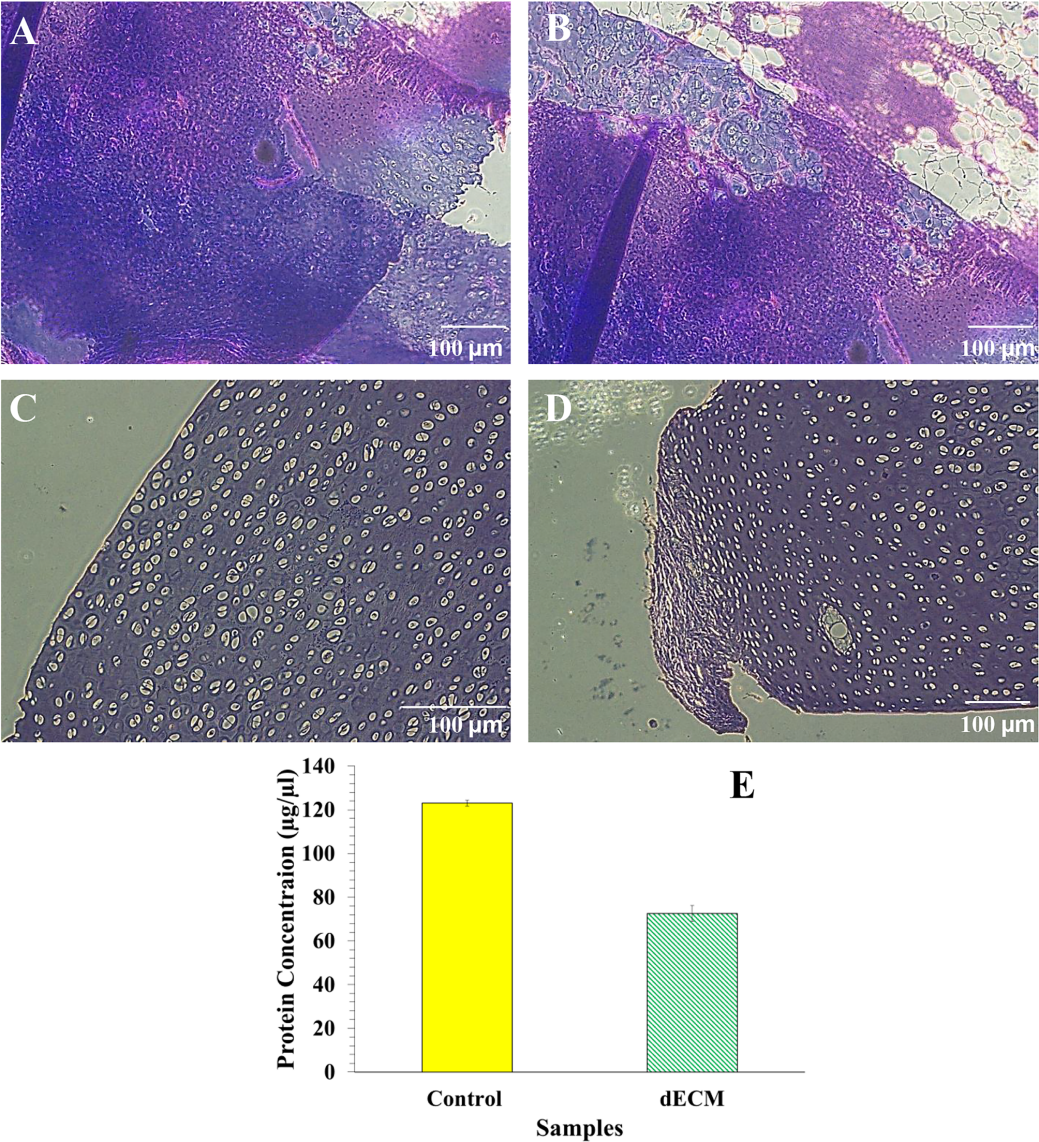
Hematoxylin and Eosin (H&E) staining of natural cartilage (**A**,** B**) and decellularized lamp scapula cartilage (**C**,** D**). Bradford results of control (natural cartilage tissue) and dECM (**E**).

Although decellularization methods may not remove all cellular components without compromising the tissue’s biochemical and mechanical properties, an acceptable level of cell removal must be ensured^21^. In this study, in order to ensure decellularization, H&E staining was performed on cartilage tissue before and after the decellularization process, and the results of these observations are shown in Fig. 1(A-D).

Based on the observed histological changes and decreased staining intensity, the decellularization process was considered successful. Chondrocytes were well removed from the cartilage tissue, and only empty lacunae cavities were seen. According to the results of decellularization, cavities are created in the tissue, which can improve cell migration, nutrient and oxygen transport, and cellular waste excretion. Freezing cycles lead to the formation of ice crystals, which lead to cell destruction and protein excretion. However, these freezing cycles have little effect on the original extracellular matrix tissue, which is an advantage of this method. Although the use of freezing cycles results in a large amount of cell destruction, cellular debris still remains in the tissue, which can lead to immunogenic reactions. Therefore, the chemical method of using sodium dodecyl sulfate has been used, which is known as the best material for removing cells and debris. Sodium dodecyl sulfate is amphipathic in nature and therefore can react with cell membranes and lead to cell membrane destruction. It can also lead to protein degradation by unfolding. However, the use of high concentrations of sodium dodecyl sulfate can lead to the destruction of collagen, which is crucial for the mechanical properties of cartilage. As a result, adjusted concentrations of sodium dodecyl sulfate are recommended for decellularization^22–24^.

### Bardford results

The Bradford assay determined the total protein concentration of the decellularized sample, and it was compared to natural cartilage tissue as the control sample (Fig. 1(E)). Decellularization led to the elimination of some points of protein concentration, while 58.98 ± 2.16% preserved the protein content^25^. It showed high preservation of protein content. Sodium dodecyl sulfate (SDS)-based decellularization led to the fast and successful removal of nuclear materials and decellularization through protein-protein interactions and solubilizes cell membrane disruption, while its concentration should be regulated to decrease biochemical damage^26^. Therefore, here two various SDS concentrations were applied and combined with physical decellularization to lower damage ECM.

### Rheology observations

Bioinks should demonstrate the shear-thinning behavior with low viscosity at the high strain phase which facilitates the extrusion procedure, enough yield stress to hold to the structure after bioprinting, and not too much G’ which prohibits bioprinting^27^.

**Fig. 2:**
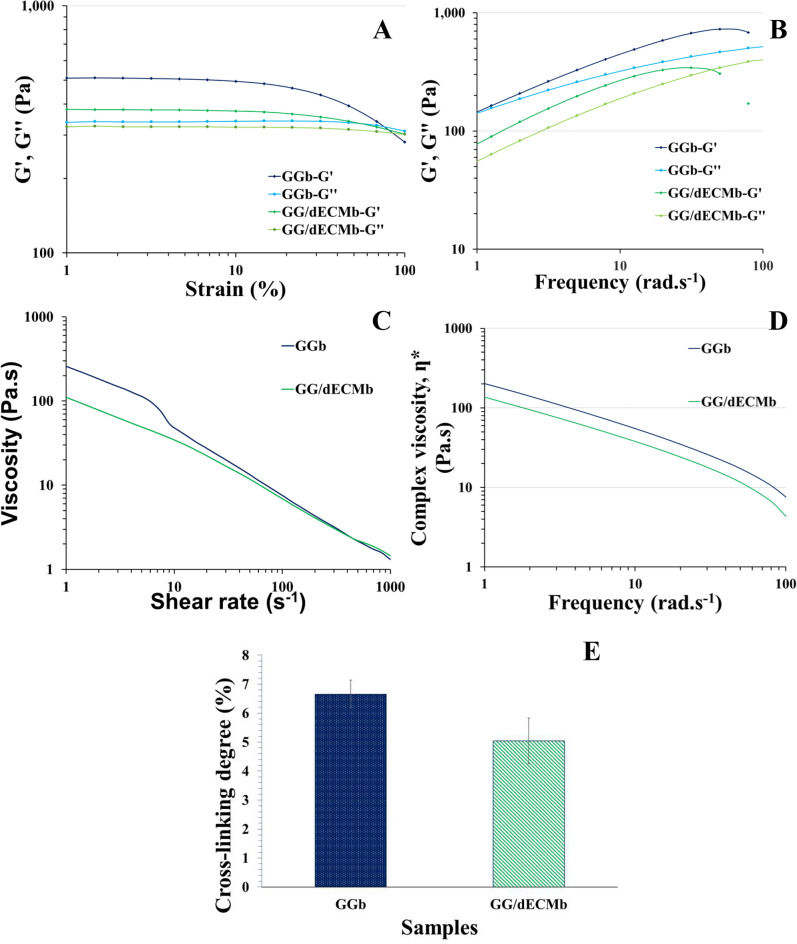
Rheological test results of GGb and GG/dECMb bioink. The strain sweep (**A**), frequency sweep (**B**), viscosity vs. shear rate curve (**C**), complex viscosity results (**D**), and the cross-linking degree (**E**) of GGb and GG/dECMb bioink.

Rheological tests were performed on the GG and dECM bioink before the 3D bioprinting procedure. The results are demonstrated in Fig. 2. According to the observations in Fig. 2(A), both bioinks had large viscoelastic regions which guaranteed the high processability of the bioinks. The other tests were performed below 10% strain in the linear viscoelastic region for both bioinks. Furthermore, the G’ was higher than G’’ which confirmed the solid-like state of the ink.

The frequency sweep test (Fig. 2(B)) was performed in the linear viscoelastic region and G’ and G’’ were detected to evaluate the viscoelastic properties. Based on the results both bioink showed the Newtonian behavior of the hydrogels with higher G’ than G’’. The higher G’ of GG demonstrated a higher solid-like of this bioink and proved its higher printability scores. According to the viscosity vs. shear rate curve (Fig. 2(C, D)), both bioinks showed viscoelastic behavior as the viscosity decreased as the shear rate increased, which is essential for 3D bioprinting.

Furthermore, the cross-linking degree was calculated from the rheological test results (Fig. 2(E)). The GG bioink showed 6.65 ± 0.48% and dECM demonstrated 5.04 ± 0.79%. The higher cross-linking degree of GG was detected in the higher solid behavior of this ink. The lower cross-linking degree of dECM bioink can be related to the higher molecular weight of GAG in this sample, which prohibited chain polymer entanglements and movements, leading to a lower chance of cross-linking^28^.

Previous studies demonstrated that the pure dECM could not be bioprinted and should have higher viscosity gels mixed with dECM to provide a bioprintable bioink with cartilage potential capabilities^29^. Therefore, here it is hopeful the mixture of GG with dECM provides a cartilage-based ink for cartilage tissue engineering. Although the dECM sample showed lower viscosity and a cross-linking degree in comparison to GG, it provided a dECM-contained ink.

### Morphology observations and printability analysis

**Fig. 3:**
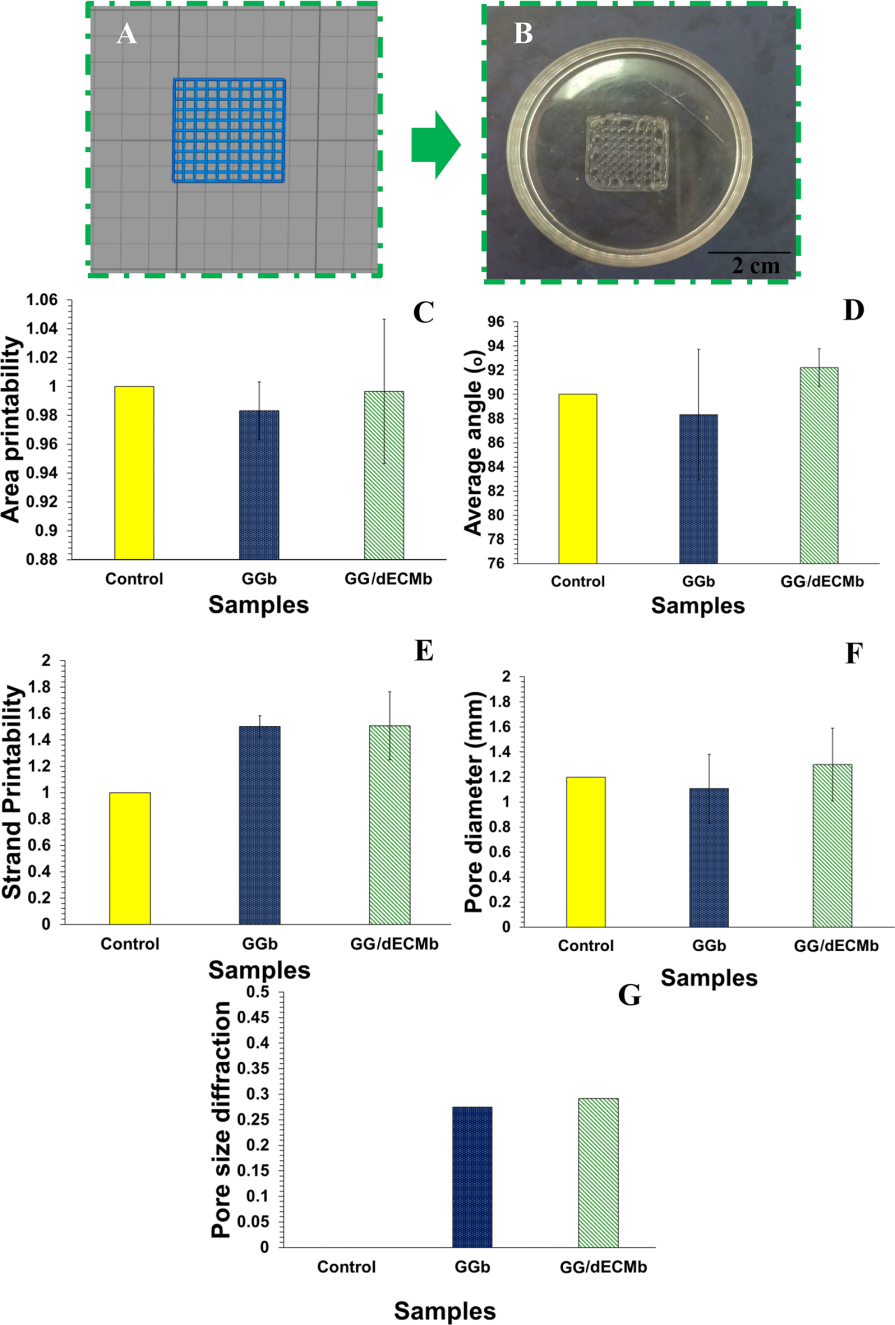
3D designed model (**A**) and 3D bioprinted (**B**) GG/dECMb cartilage tissue engineering scaffold. Printability characterization of GG/dECMb scaffold in comparison to GGb scaffold and 3D control design; area printability (**C**), angle accuracy (**D**), strand printability (**E**), pore diameter printability (**F**), and pore size diffraction (**G**).

The scaffolds were designed using SolidWorks software (Fig. 3(A)), and then the bioprinting process was carried out. In the 3D bioprinting process, although high precision is used in the design of scaffolds, scaffolds are usually not created exactly as intended, and differences are seen in the size or structure of the pores, strands, or entire structure. Therefore, measuring the printability index is of great importance in the printing process. In general, printability means the ability of the scaffold to create and maintain the structure after printing based on the design^30^.

The morphology observation of the GG/dECMb scaffold is shown in Fig. 3(B). In this study, the printability of bioinks was examined based on 5 parameters: area printability (A), angle accuracy (B), strand printability (C), pore diameter printability (D), and pore size diffraction (E), and the results are demonstrated in Fig. 3(C-G), respectively. Based on the achieved results, both GGb and GG/dECMb scaffolds showed high printability, which was close to the designed scaffold. In terms of area printability, the dECMb scaffold had higher precision, and the obtained structure was close to the designed scaffold. In the angle accuracy index, both scaffolds were slightly different from the designed scaffold. Although GGb had a near contact angle to control, which can be due to its higher cross-linking, and as a result, more consistency.

In terms of strand diameter, the two scaffolds performed almost the same. However, in terms of pore size and pore size distribution, the GGb scaffold performed better. Although there were some differences in printability parameters, both scaffolds showed high printability and saved the structure. It seems both constructs could serve as cartilage tissue engineering scaffolds, which were investigated more in vitro.

### Fourier transform infrared spectroscopy (FTIR)

**Fig. 4:**
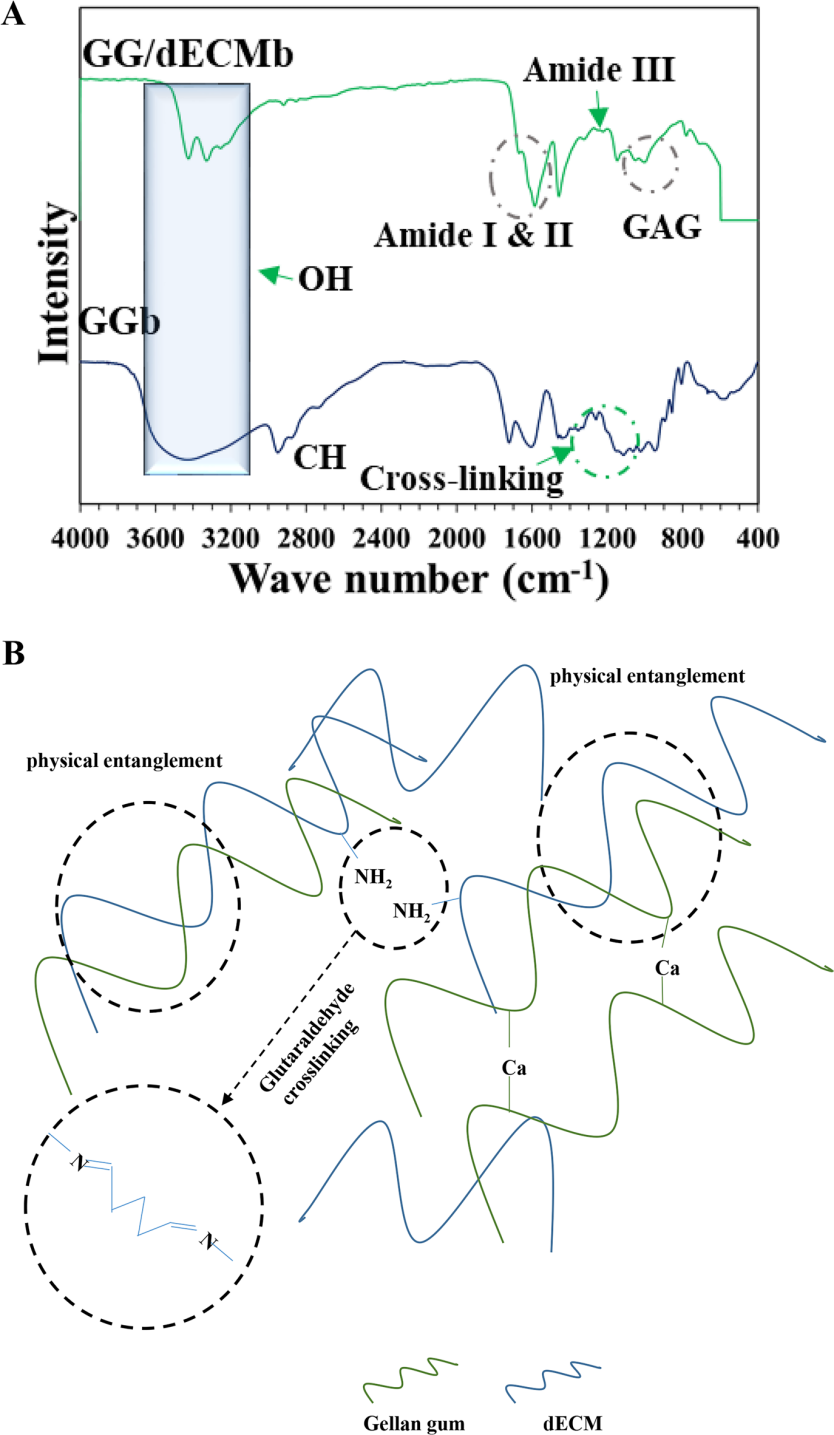
FTIR image of GG and dECM scaffolds (**A**). The schematic of proposed chemical and cross-linking reactions (**B**).

To chemical investigation of the samples, an FTIR test was performed on both GGb and GGb/dECM scaffolds (Fig. 4 (A)). OH stretching was detected at around 3400 cm⁻¹. The -C = O groups and the symmetric stretching of anionic carboxylate groups peaks were detected at 1570 cm⁻¹ and 1460 cm⁻¹, respectively. C-H stretching at 2922 cm⁻¹ was observed. Cross-linking led to the formation of COO- peak at 1278 cm⁻¹, 1180 cm⁻¹, and 1022 cm⁻¹^31,32^. After dECM addition GAG related peak was seen at 1048 cm⁻¹^33^. Furthermore, this peak can be related to the hydrogen bonding and interaction between the hydroxyl group of gellan gum with proteins, peptides, or polysaccharides in dECM^34,35^. In addition, dECM-related peaks were detected with low intensity at 1640 (amide I, C = O bond), 1580 (amide II N-H and C-N bonds), and 1245 amide III (C-N bond)^36,37^. Therefore, it seems, that dECM was successfully incorporated in the GGb/dECMb sample. Crosslinking-related peaks were detected with lower intensity, which can be considered a sign of a lower cross-linking degree. The schematic of proposed chemical and cross-linking reactions is shown in Fig. 4(B).

### Mechanical results

**Fig. 5:**
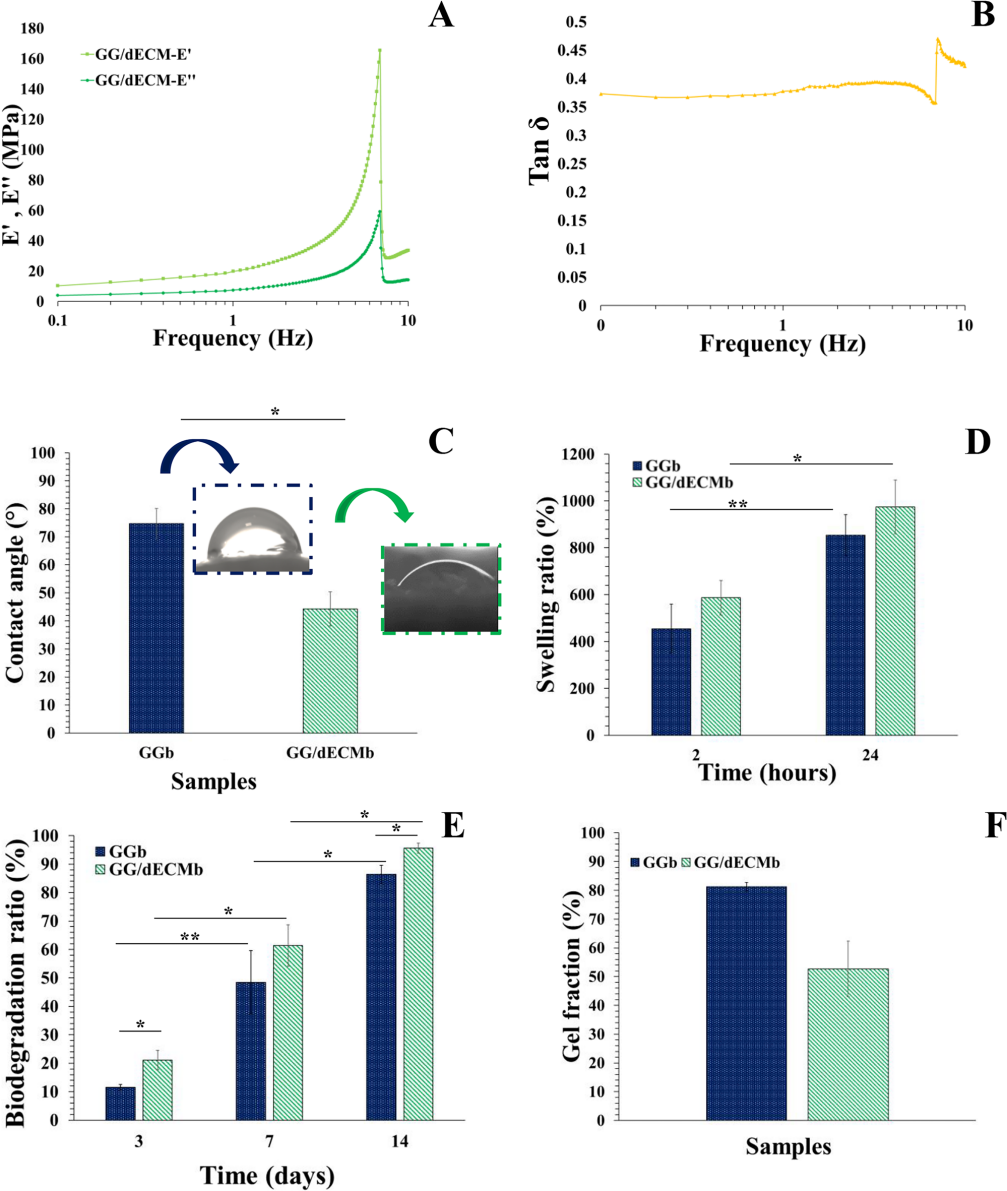
Storage modulus (E’) and loss modulus (E’’) as a function of frequency (**A**), the tan δ (**B**), contact angle (**C**), swelling ratio during 24 h soaking in PBS solution (**D**), biodegradation ratio during 14 days (**E**), and gel fraction (**F**) of GGb and GG/dECMb scaffolds.

As the cartilage ECM has a crucial role in the biomechanical properties, distributing pressure and shear stress to facilitate joint motion, the cartilage scaffold mechanical properties are important^34^. Therefore, the dynamic mechanical test was carried out, and the results were demonstrated in Fig. 5(A). The storage modulus (E’) was almost constant until 1 Hz. An increase was seen at 1 to 10 Hz. For loss modulus (E’’), a similar trend was seen, constant between 0.1 and 1 Hz, and there was an increase from 1 to 10 Hz. This increase in the E’’ could demonstrate that this scaffold can dampen some of the mechanical vibrations due to its viscoelastic nature^38^. The viscoelastic nature of hydrogels is crucial in cartilage tissue engineering because it allows them to mimic the mechanical properties of natural cartilage, which is essential for cell behavior and tissue regeneration^39^.

The tan δ (loss factor) is depicted in Fig. 5(B). As can be seen, this sample showed a tan δ higher than 0.3, which confirmed the viscoelastic behavior of the scaffold. The loss factor indicates the ratio of the energy dissipating and demonstrates the capability of the material to dampen. Increasing the tan δ by increasing the frequency means that the scaffold is highly viscous and less viscous at high frequencies. Although it has some capacity, its behavior continues near 10 Hz and at high frequency, leading to structural dysfunction^40^.

### Contact angle, swelling ratio, and degradation

The hydrophilicity of the samples and their interaction with body fluid is another important criterion that affects cellular adhesion and, as a result, tissue regeneration. Usually, the oxygen-containing groups enhance water molecules’ interaction with the scaffold surface and lead to contact angle reduction^41^. Based on previous studies, pure gellan gum is a hydrophilic polymer, and its carboxyl orientation has a lot of effect on its interaction with water molecules^42^.

Here, the GG sample demonstrated a 74.73 ± 5.4° contact angle (Fig. 5(C)). Although glutaraldehyde cross-linking increased its contact angle, due to previous studies^43^. While the addition of dECM reduced the contact angle of the GG/dECMb scaffold to 44.27 ± 6.0°. This can be due to dECM composition. Cartilage ECM typically contained type II collagen and the sulfated proteoglycan, aggrecan^44^. Therefore, our results were in line with a previous study, which demonstrated that the addition of ECM to the scaffold decreased the contact angle and improved the hydrophilicity^45^. Furthermore, the rheological results confirm a slightly lower cross-linking degree of this sample, which can facilitate the water molecule penetration and decrease the contact angle. The resulting contact angle of the GG/dECMb scaffold is in the suitable range for chondrocyte adhesion and proliferation^46^.

For further evaluation of the fluid-scaffold interaction, the swelling capacity of the scaffolds was evaluated, and the results are shown in Fig. 5(D). The structure of the scaffold, surface interactions, and cross-linking degree are effective in swelling capacity^47^. The swelling capacity was increased after the addition of dECM in the bioink and achieved 587.05 ± 73.61% and 974.37 ± 114.30 after 2 and 24 h immersion in PBS solution, respectively, while usually the addition of cartilage-based dECM concentration increment led to lower swelling capacity. This swelling capacity increase can be due to the slight cross-linking degree. Furthermore, the contact angle is the factor in the water absorption and water retention, and the more hydrophilic nature of the GG/dECMb sample can be effective in its higher swelling capacity.

Degradation is known as one of the effective behaviors of the scaffolds, which should be studied to evaluate the scaffolds’ efficiency. It can impact cell behaviors such as adhesion, viability, and growth. The degradation ratio should be matched with tissue regeneration; a low degradation ratio hampers proliferation, and fast degradation could not provide enough mechanical support for tissue regeneration^48,49^. The results of the degradation evaluation are demonstrated in Fig. 5(E). According to the results, both samples were degradable, and most of the structures were degraded during the 14 days. At each time point, the GG/dECMb sample showed a slightly higher degradation ratio, and it almost completely degraded after 14 days (95.60 ± 1.74%). A high swelling ratio leads to faster degradation; on the other hand, the higher contact angle provides lower hydrophilicity and therefore limits the fluid penetration into the structure and reduces the swelling ratio and, as a result, the biodegradation ratio^50^.

In fact, although both scaffolds’ degradation is fast compared to low-speed cartilage regeneration, based on a previous study’s results, which evaluated gellan-gum scaffolds in PBS and simulated body fluid degradation, GG scaffolds showed a lower degradation ratio in the simulated body fluid due to the presence of ions and their interactions with gellan-gum-based scaffolds, which provide stiffer and higher mechanical properties scaffolds with a lower degradation ratio^51^.

### Gel fraction

The gel fraction of GGb and GG/dECMb scaffolds was evaluated, and the results are shown in Fig. 5(F). According to the results, GG/dECMb showed a slightly lower gel fraction. These results were not far from expected. Because gel fraction is related to the cross-linking reactions of the scaffolds and the rheological evaluations, the cross-linking degree demonstrated a lower cross-linking degree for the GG/dECMb sample compared to the GGb preprint. As can be seen, the bioprinting procedure has not had a great impact in this manner, and GGb has a higher gel faction ratio after bioprinting (81.20 ± 1.4%). Furthermore, the contact angle and swelling ratio evaluations demonstrated higher hydrophilicity and fluid penetration in the GG/dECMb sample; therefore, it suggested that some parts of this scaffold can be degraded under the gel fraction test conditions that could be the reason for 52.61 ± 9.67% gel fraction of GG/dECMb sample.

### Cellular investigations

**Fig. 6:**
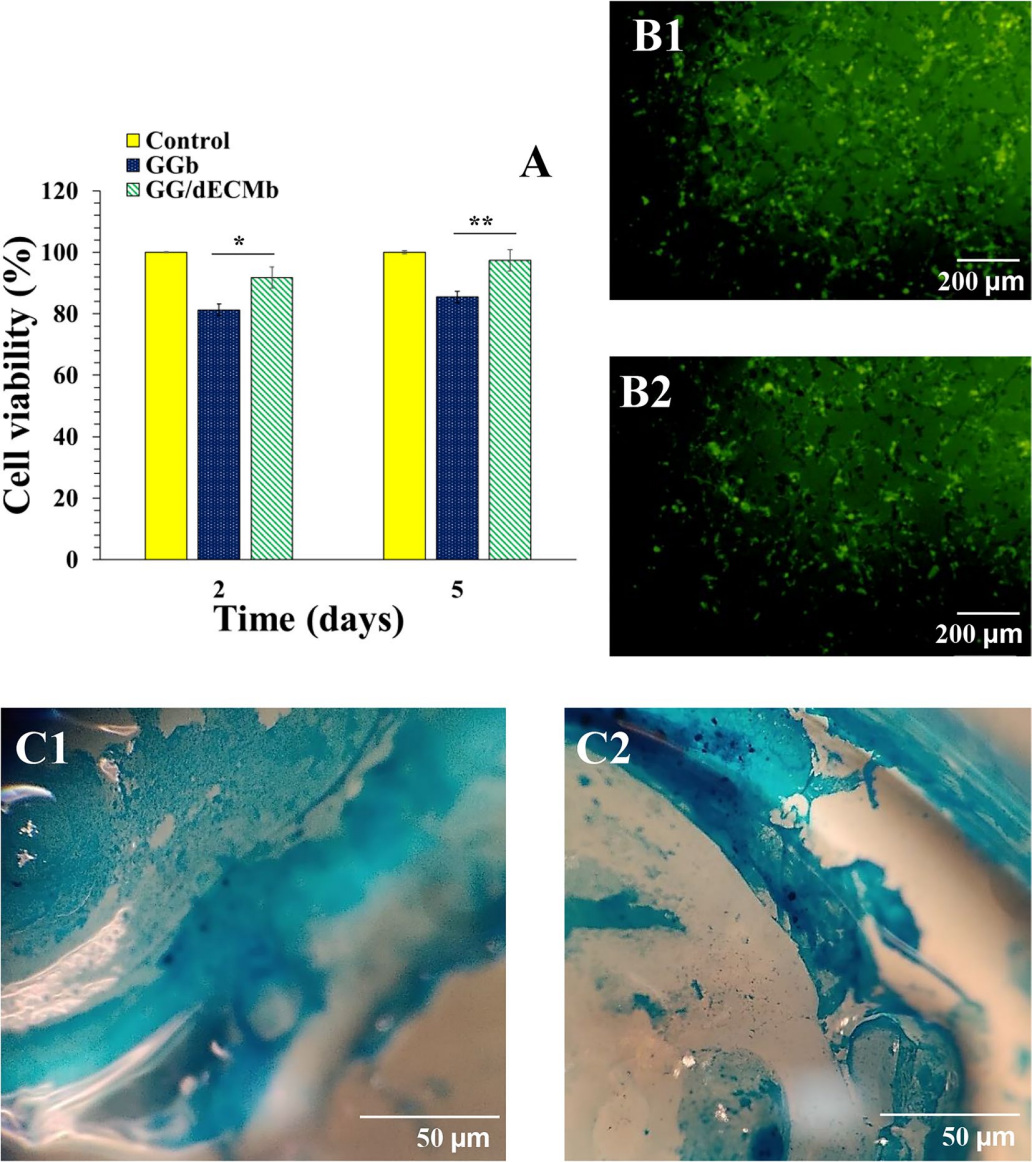
Cell viability using the MTT test after 2 and 5 days of cell culturing on GGb and GG/dECMb scaffolds (**A**). Live/dead observations after 2 days (**B1**,** B2**) and Alcian blue staining after 21 days (**C1**,** C2**) on the GG/dECMb scaffold.

The success of a scaffold in tissue regeneration depends on various aspects, including morphological, physicochemical, and cellular behaviors. The cellular viability on these scaffolds was further evaluated by the 3-[4,5-dimethylthiazol-2-yl]−2,5 diphenyl tetrazolium bromide (MTT) test, and the results are demonstrated in Fig. 6(A). According to the test, both GG and GG/dECMb scaffolds showed high cell viability after 2 and 5 days, and the cell viability was increased during 2 to 5 days for both scaffolds.

In comparison, GG/dECMb scaffolds with higher cell-scaffold interactions and cell viability reaching 97.41 ± 1.02% after 5 days, while GGb showed 85.44 ± 1.54%, Although GGb scaffold also showed high cell viability, while the higher cell viability on the GG/dECM scaffold it can be related to the dECM components in the scaffolds, which provide more suitable environment for cell adhesion and interactions.

To further investigate if the samples were cell-friendly, the live/dead test was performed on the GG/dECMb sample, and the results are shown in Fig. 6(B1 and B2). Based on the results, all the cells were alive and detected with green dyes. There was no sign of red points, which showed dead cells. This also proved the high potential of the GG/dECMb scaffold in supporting cell viability.

In addition, Alcian blue staining was performed on the GG/dECMb sample to confirm the cultured cells’ differentiation capability to repair articular cartilage. The results are shown in Fig. 6(C1 and C2). Based on the observation, Alcian blue staining after 21 days of culturing indicated that the glycosaminoglycans deposition can be considered a sign of successful differentiation to chondrocytes.

Overall, cellular investigations showed that pure gellan gum demonstrated high cell-friendly behavior, while the most important part in the improvement of cell interactions is cartilage dECM. Urea-extracted dECM usually has a high concentration of small and moderate molecular weight proteins^20^. These results were in line with a previous study that demonstrated that the use of urea extraction dECM could enhance chondrogenic activity^52^.

## Materials and methods

### Materials

Gellan gum (Sigma-Aldrich, St. Louis, MO, USA), calcium chloride (CaCl₂, Sigma-Aldrich, St. Louis, MO, USA), and glutaraldehyde (25%, Sigma-Aldrich, St. Louis, MO, USA) were used for bioink preparation. Phosphate buffer saline (PBS, dry powder) was purchased from Sigma-Aldrich, St. Louis, MO, USA. Aqueous solutions were prepared with deionized water. For decellularization, sodium dodecyl sulfate (SDS), urea, and sodium chloride (NaCl, > 99.99%) were obtained from Sigma-Aldrich.

### Tissue harvest and decellularization procedure

Lamb scapula from 6- to 12-month-old lambs was supplied from local abattoirs. The cartilage samples were rinsed with phosphate-buffered saline (PBS), and any fatty parts and excess tissues were carefully removed. The samples were then cut into small pieces, rinsed again with PBS, and transferred to a freezer at −20 °C for further use.

The decellularization procedure was performed using a combination of physical and chemical phases. Initially, the samples underwent five cycles of freezing using liquid nitrogen for 2 min each, followed by washing with PBS and refreezing with liquid nitrogen. Freeze-thaw cycles cause disruption of cell membranes and induce apoptosis in chondrocytes^53^. Afterward, they were immersed in a 2% SDS solution for 3–5 h with gentle stirring. After removal from the solution, the samples were washed three times with deionized water. They were then immersed again in a 4% SDS solution for 1–3 h. Finally, the matrices were taken out of the solution and washed five times with deionized water^54^.

### Urea extraction

Urea-extracted dECM can retain the bioactivities of ECM and improve cell proliferation, migration, and differentiation^55^. Therefore, urea extraction was performed to prepare solubilized dECM with 2 M urea in a 150 mM NaCl solution at 4 °C for 48 h. Then, centrifugation was conducted to separate the insoluble particles at 2000 rpm for 5 min. The supernatant was dialyzed against PBS for 24 h^55,56^.

### Bioink preparation

For bioink preparation, the pre-crosslinking method was used through ionic crosslinking using the Ca^2+^ ions present in calcium chloride, as well as the covalent crosslinking process using glutaraldehyde^57–59^. An extracellular matrix solution was prepared with 0.5% (w/v) calcium chloride in deionized water and stirred for 30 min. After this period, to prepare a 3% gellan gum solution, the gellan gum powder was weighed and added to the beaker. The mixture was then stirred for an additional 2 h at 60 °C to ensure complete dissolution and a uniform solution. To promote dual cross-linking, 2% (v/v) glutaraldehyde was added to the solution, followed by stirring for another 6 h to ensure thorough mixing and complete dissolution. The bioink composed of gellan gum and decellularized extracellular matrix was abbreviated as GG/dECMb for convenience throughout the manuscript. The control bioink containing gellan gum-free dECM bioink was also prepared in deionized water for further investigation and named gellan gum bioink (GGb).

### Bioprinting of scaffolds

**Fig. 7:**
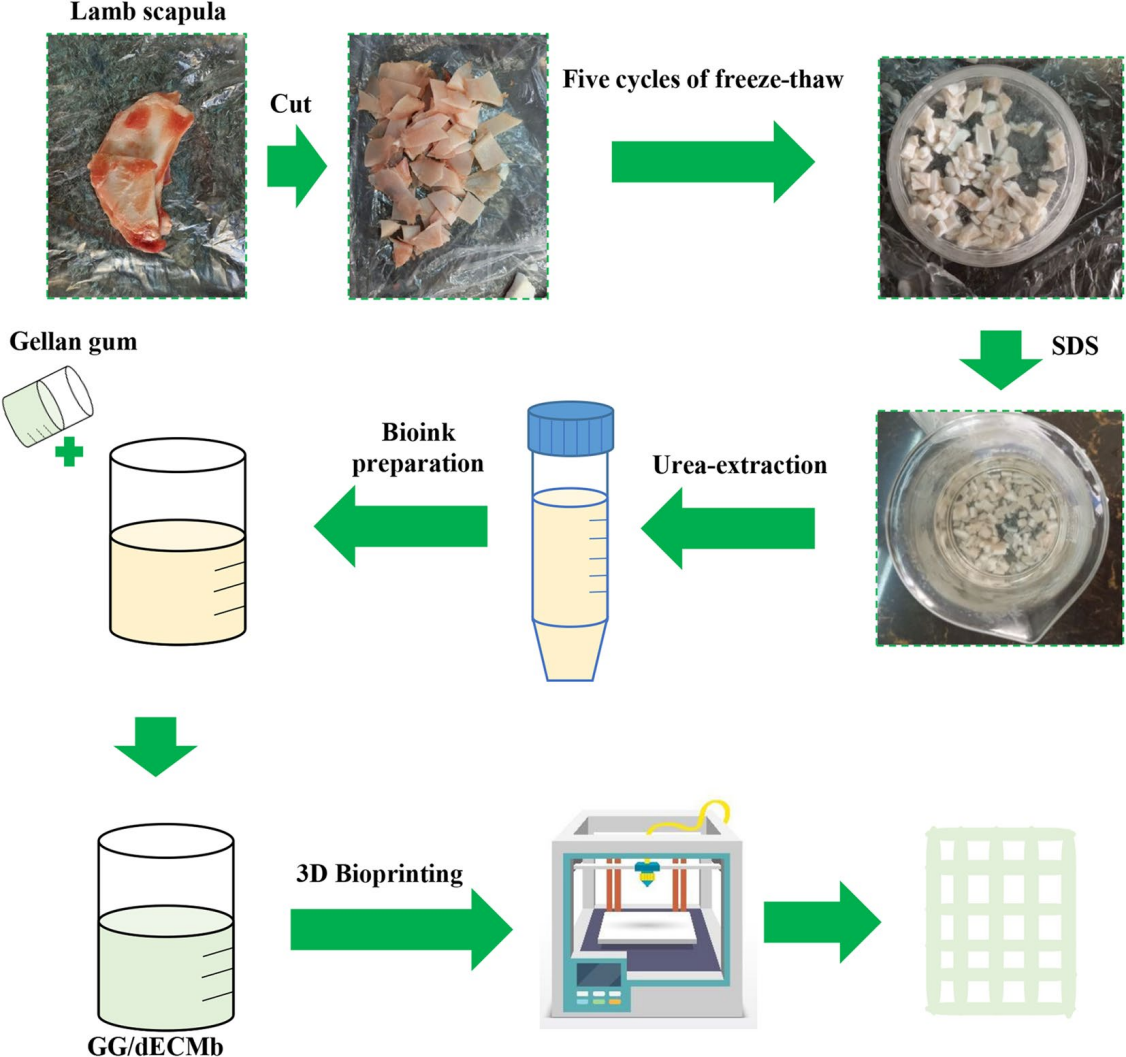
Schematic of GG/deCMb scaffold preparation steps.

The bioprinting step was conducted using an extrusion-based 3D bioprinter (3D BIOPRINTER N2, 3DPL Co. Ltd., Tehran, Iran). For this purpose, the scaffold was designed using SolidWorks software (2016 version) (20 × 20 mm and consisted of 10 × 10 grid structure with strand diameters of approximately 0.4 mm. A total of 4 layers were printed with a layer height of 300 μm, resulting in an estimated infill density of ~ 53%). The prepared bioinks were transferred to the bioprinting tube with a 21G stainless steel nozzle. The bioprinting procedure was performed at 0.8–1.5 kPa and 10 mm/s at 25 °C. The schematic of GG/deCMb scaffold preparation steps is depicted in Fig. 7.

### Characterizations

#### Hematoxylin and Eosin (H&E)

To verify the decellularization procedure’s success, H & E staining was performed on the dECM samples before the urea extraction process. The native lamb scapula cartilage was considered as the control sample. To carry out the samples, they were dehydrated with 70% ethyl alcohol fixed with xylene (Merck) and embedded in paraffin. Then, they were sliced using a microtome and stained with H & E. The tissue sections were observed under a microscope.

#### Bradford

The protein concentration in the extracted dECM was evaluated by Bradford protein assay and compared to natural cartilage samples. For this purpose, 180 µl of Bradford reagent was added to each ELISA plate, and 20 µl of standard solutions and/or urea-extracted dECM solution were added to each well plate and mixed well using pipetting. The samples were incubated for 5 min, and the optical density (OD) was measured at 595 nm with a plate reader.

#### Morphology observations and printability

The morphology of bioprinted scaffolds was observed using a digital camera (5 MP). Furthermore, the bioprintability of the bioinks was assessed using an investigation of five printing parameters, including area printability, strand printability, angle accuracy, pore size bioprintability, and pore size accuracy. For area printability evaluation, the area of the bioprinted structure (A_1_) was compared to the designed scaffold (A_0_), and area printability was calculated using Eq. 1.

1 $$\rm Area\ printability = (A0/A1)$$

The same, for strand printability evaluation, the strand of the designed structure was assumed as D_0_, the printed structure was considered as D_1_, and the strand printability was calculated using Eq. 2.

2 $$\rm Strand\ printability = 1- (D0-D1/D1)$$

For angle accuracy, pore size printability, and pore size accuracy, the angle and pore size of the printed scaffold were compared to the designed scaffold, and for the pore size accuracy evaluation, its diffraction from the ideal pore size based on the designed scaffold was considered. These measurements were performed using the image analysis program ImageJ (US National Institute of Health, Bethesda, MD).

#### FTIR

The composition of the scaffolds was evaluated using a Fourier transform infrared spectrophotometer (FTIR, Nicolet Is10, Thermo Fisher Scientific, MA, USA). FTIR spectra demonstrated an average of 30 scans between 400 and 4000 cm⁻¹ at a resolution of 10 cm⁻¹.

#### Rheological

For the rheological measurements, a Physica MCR301 rheometer (Anton Paar, Austria) was used at room temperature. Strain sweep, frequency sweep tests, and shear rate sweep tests were conducted on bioinks before bioprinting. In the strain sweep test, the stain was applied at 0.01–100% at 10 rad.s⁻¹ angular frequency. The frequency sweep was performed in the range of 0.05–500 rad/s and 10% strain. The storage modulus (G’), loss modulus (G”), tan δ, and complex viscosity were calculated using the frequency sweep test. In addition, viscosity was reported at shear rates of 1–1000 s⁻¹ shear rate.

In addition, the cross-linking degree was calculated using the reported storage modulus, which was found in the linear region of hydrogel behavior. The cross-linking degree was calculated using Eq. 3^60^.

3 $$\rm Cross-linking\ degree\ (\%) = G^\prime/3RT*100$$

where T stands for temperature in kelvin, R is the gas constant (J/(mol·K)), and G’ is the storage modulus (Pa)^60^.

#### Contact angle, swelling, and biodegradation

The hydrophilicity of the scaffolds was assessed by measuring the contact angle using the sessile drop method with distilled water. For this purpose, a water drop was dropped on the surface of the scaffolds, and the angle between the drop and the surface was measured. The test was repeated three times, and the average was considered as the contact angle.

To evaluate the capability of the scaffold to interact with fluids and measure swelling capability, the scaffolds were immersed in a falcon containing 30 ml PBS solution and transferred to a thermoshaker at 37 °C for 24 h. The dry weight of the dried scaffolds before soaking was considered as W_0_, and the wet weight after 2 h and 24 h was considered as W_1_. The swelling capacity was measured using Eq. 4^61^.

4 $$\rm Swelling\ ratio (\%) = [(W1-W0)/W0]*100$$

Furthermore, the degradation ratio of the scaffolds was measured to investigate the degradability behavior of the scaffolds. For this purpose, the dry scaffolds were weighted (W_1_) and were soaked in PBS solution for 2 weeks. The air-dried scaffold weights were measured in intervals (3, 7, and 14 days) and considered as W_2_. The degradation ratio was measured using Eq. 5^62^.

5 $$\rm Degradation\ ratio (\%) = [(W1-W2)/W1]*100$$

#### Gel fraction

Gel fraction measurements were carried out after 7 h of immersion in deionized water immersion of the dried scaffolds. Dry weights before and after 7-hour immersion at room temperature (W₀ and W₁ respectively) were recorded. The gel fraction was measured using Eq. 6^63^.

6 $$\rm Gel\ fraction(\%) = [(W1)/ W0]*100$$

#### Mechanical evaluation

The viscoelastic behavior of the sample in tension mode was evaluated using dynamic mechanical thermal analysis (DMTA). A rectangular film measuring 10.00 mm × 5.00 mm × 0.30 mm was prepared for the test. The rectangular film was prepared by casting the hydrogel solution into silicone molds and then dried at room temperature to a uniform thickness. The frequency sweep test was conducted at 37 °C, and the frequency was changed from 0.1 to 10 Hz. All measurements were carried out in the linear viscoelastic region. The storage modulus (E’), loss modulus (E”), and mechanical damping parameter (tan δ) were determined^64^.

#### Cellular investigations

##### MTT

Rat mesenchymal stem cells were supplied from the Pasteur Institute of Iran. The scaffolds were sterilized with repeated PBS washing and then exposed to UV for 1 h. To investigate the capability of the scaffolds to support cell viability and proliferation, an MTT test was performed on the scaffolds. The sterilized dried scaffolds were seeded with 1 × 10⁴ cells and incubated at 37 ± 0.5 °C in 95% humidity and 5% CO₂ for 2 and 5 days. After each interval, the samples were washed with PBS, and 0.5 mg/mL of MTT was added and incubated for 5 h. Then, dimethyl sulfoxide was added to dissolve formazan crystals, and the absorbance was measured at 570 nm using an ELISA reader.

##### Live/dead

To ensure the cell viability of the cultured cells on the dried scaffolds, a live/dead investigation was performed. For this, the 1 × 10⁴ rat mesenchymal stem cells were cultured on the scaffolds, and after 2 days, the samples were washed with PBS, and the cells were fixed. Then a staining process was performed for 30 min using calcein-AM (green) and ethidium homodimer-1 (red) dyes, which demonstrated green and red cells, respectively. The samples were observed under an inverted fluorescence microscope.

##### Alchian

In order to evaluate the chondrogenic differentiation of the dried scaffolds, Alcian blue staining was performed. For this, the scaffolds were cultured with 3 × 10⁵ rat mesenchymal stem cells in Dulbecco’s Modified Eagle Medium/F12 medium supplemented with 10% FBS and 1% penicillin-streptomycin for 4 h. After the first seeding procedure step, the medium was removed and replaced with a chondrogenic differentiation medium. The culture lasted for 21 days, and every 3 days the culture medium was refreshed.

After this duration, the scaffolds were fixed with formalin (10%) for 3 min, followed by the rising step. Then they were stained with alcian blue 1% in 3% acetic acid at pH 2.5 for 30 min. The samples were observed under a microscope.

##### Statistical analysis

All the tests were repeated at least three times, and the results were reported as the mean ± standard deviation. Data were collected in Microsoft Excel 2016 software, and the statistical significance of the average values was calculated using a two-sample t-test in Microsoft Excel 2016 software, and a p-value of ≤ 0.05 was considered statistically significant (as p-value ≤ 0.05 and *p* < 0.001 (**)).

## Conclusion

Finding the most suitable substitute for cartilage tissue regeneration is vital and challenging. Previously, numerous studies evaluated various hydrogels for cartilage tissue engineering while optimizing the compounds will be continuous. Here, the novel GG/dECMb compound was formulated, 3D bioprinted, and evaluated with different in vitro tests. Based on the results, gellan gum is a suitable suggested hydrogel for cartilage regeneration, while it has low cellular interactions and pure dECM, although it shows high potential for cellular interaction, has low mechanical properties, and has limited printability. The dECM addition to gellan gum and prepared GG/dECMb scaffold increased the hydrophilicity and water-scaffold interaction of the GGb scaffold. The GG/dECMb scaffold showed improved mechanical properties for cartilage repair. Furthermore, the addition of dECM to this hydrogel improved cellular viability, proliferation, and differentiation and is more suitable for cartilage tissue engineering. Further, in vivo tests were suggested to be carried out in the future to confirm this compound for cartilage repair.

## Data Availability

The data that support the findings of this study are available from the corresponding author upon reasonable request.
